# Supplementary material for: Resolving the full spectrum of human genome variation using Linked-Reads
Source: Genome Res. 2019 Apr;29(4):635–45. doi: 10.1101/gr.234443.118 (PMC6442396; doi:10.1101/gr.234443.118)
Supplement: Supplemental Material [file supp_gr.234443.118_Supplemental_longranger-2.2.zip › longranger-2.2.tar.bz2!/lr2.2-public/lib/stan/cmdstan/stan/doc/api/html/accumulator_8hpp_source.html]

Stan: src/stan/math/matrix/accumulator.hpp Source File


|  |  |
| --- | --- |
|  | Stan  2.5.0  probability, sampling & optimization |


- Main Page
- Related Pages
- Namespaces
- Classes
- Files

- File List
- File Members

All Classes Namespaces Files Functions Variables Typedefs Enumerations Enumerator Friends Macros Pages

- src
- stan
- math
- matrix

accumulator.hpp

Go to the documentation of this file.

1 #ifndef STAN\_\_MATH\_\_MATRIX\_\_ACCUMULATOR\_HPP

2 #define STAN\_\_MATH\_\_MATRIX\_\_ACCUMULATOR\_HPP

3

4 #include <vector>

5 #include <boost/utility/enable\_if.hpp>

6 #include <boost/type\_traits/is\_arithmetic.hpp>

7 #include <boost/type\_traits/is\_same.hpp>

8 #include <stan/math/matrix/Eigen.hpp>

9 #include <stan/math/matrix/sum.hpp>

10

11 namespace stan {

12  namespace math {

13

24  template <typename T>

25  class accumulator {

26  private:

27  std::vector<T> buf\_;

28

29  public:

33  accumulator()

34  : buf\_() {

35  }

36

40  ~accumulator() { }

41

52  template <typename S>

53  typename boost::enable\_if<boost::is\_arithmetic<S>, void>::type

54  add(S x) {

55  buf\_.push\_back(static\_cast<T>(x));

56  }

57

70  template <typename S>

71  typename boost::disable\_if<boost::is\_arithmetic<S>,

72  typename boost::enable\_if<boost::is\_same<S,T>,

73  void>::type >::type

74  add(const S& x) {

75  buf\_.push\_back(x);

76  }

77

87  template <typename S, int R, int C>

88  void add(const Eigen::Matrix<S,R,C>& m) {

89  for (int i = 0; i < m.size(); ++i)

90  add(m(i));

91  }

92

102  template <typename S>

103  void add(const std::vector<S>& xs) {

104  for (size\_t i = 0; i < xs.size(); ++i)

105  add(xs[i]);

106  }

107

113  T sum() const {

114  using math::sum;

115  return sum(buf\_);

116  }

117

118  };

119

120

121

122

123  }

124 }

125

126 #endif

stan::math::accumulator::sum

T sum() const

Return the sum of the accumulated values.

**Definition:** accumulator.hpp:113

stan::math::accumulator::add

boost::disable\_if< boost::is\_arithmetic< S >, typename boost::enable\_if< boost::is\_same< S, T >, void >::type >::type add(const S &x)

Add the specified non-arithmetic value to the buffer.

**Definition:** accumulator.hpp:74

stan::math::accumulator::accumulator

accumulator()

Construct an accumulator.

**Definition:** accumulator.hpp:33

Eigen.hpp

stan::math::sum

double sum(std::vector< double > &x)

**Definition:** sum.hpp:10

stan::math::accumulator

Class to accumulate values and eventually return their sum.

**Definition:** accumulator.hpp:25

stan::math::accumulator::add

void add(const std::vector< S > &xs)

Recursively add each entry in the specified standard vector to the buffer.

**Definition:** accumulator.hpp:103

stan::math::accumulator::add

void add(const Eigen::Matrix< S, R, C > &m)

Add each entry in the specified matrix, vector, or row vector of values to the buffer.

**Definition:** accumulator.hpp:88

stan::math::accumulator::~accumulator

~accumulator()

Destroy an accumulator.

**Definition:** accumulator.hpp:40

sum.hpp

stan::math::accumulator::add

boost::enable\_if< boost::is\_arithmetic< S >, void >::type add(S x)

Add the specified arithmetic type value to the buffer after static casting it to the class type T...

**Definition:** accumulator.hpp:54


---

[ Stan Home Page ]

*© 2011–2014,
Stan Development Team.*
